# Comparative Effects of Home-Based and Aquatic Resistance Training on Hand Tremor Severity and Manual Dexterity in Older Adults with Essential Tremor: A Preliminary Randomized Controlled Trial

**DOI:** 10.3390/life16020218

**Published:** 2026-01-28

**Authors:** Cemal Polat, Tuba Sevil, Zarife Pancar, Luca Russo

**Affiliations:** 1 Department of Coaching Education, Faculty of Sports Sciences, Eskişehir Technical University, 26555 Eskişehir, Turkey; cpolat@eskisehir.edu.tr; 2 Department of Recreation, Faculty of Sports Sciences, Eskişehir Technical University, 26555 Eskişehir, Turkey; 3 Department of Physical Education and Sports, Faculty of Sports Science, Gaziantep University, 27310 Gaziantep, Turkey; 4 Department of Theoretical and Applied Sciences, eCampus University, Via Isimbardi 10, 22060 Novedrate, Italy; luca.russo2@uniecampus.it

**Keywords:** manual dexterity, aquatic exercise, essential tremor, resistance training

## Abstract

Essential tremor (ET) negatively affects neuromuscular control and hand function in older adults. Resistance exercise may enhance musculoskeletal and functional capacity, yet its modality-specific effects in ET remain unclear. This study compared the effects of home-based and aquatic resistance training on tremor severity, manual dexterity, and handgrip strength in older adults with ET. Twenty-seven participants were randomly assigned using block randomization to a home-based resistance exercise group (HBREG; n = 9), an aquatic resistance exercise group (AREG; n = 9), or a control group (CG; n = 9). Both intervention groups completed an 18-session resistance exercise program, with initial sessions supervised and subsequent sessions performed independently under regular monitoring. Tremor severity (FTMTRS), manual dexterity (Nine-Hole Peg Test), and handgrip strength were assessed pre- and post-intervention. Within-group changes were analyzed using the Wilcoxon signed-rank test and between-group differences using the Kruskal–Wallis test with Bonferroni-adjusted Mann–Whitney U tests (*p* < 0.05). Both HBREG and AREG demonstrated significant improvements in drawing and pouring tremor tasks, manual dexterity, and handgrip strength compared with the control group, with large effect sizes across outcomes. No significant differences were observed between the two exercise modalities, and no improvement occurred in the highest-difficulty spiral-B task. These findings indicate that both home-based and aquatic resistance training are safe and effective non-pharmacological strategies for reducing tremor severity and enhancing upper-extremity function in older adults with ET.

## 1. Introduction

Changes in upper-limb motor skills are commonly observed during the normal ageing process [1]. One contributing factor to this decline is the age-related increase in tremor amplitude [2,3,4], which can impair fine motor control and functional task performance in older adults. Tremor, defined as an involuntary rhythmic oscillatory movement of a body part, is the most common movement disorder and may occur in one or multiple anatomical regions. Notably, up to 94% of tremor cases involve the hands, underscoring its impact on upper-extremity function. Essential tremor is one of the most prevalent tremor syndromes and neurological movement disorders [5], with a reported prevalence of approximately 4% in older adults [6] and 3.09% (95% CI: 2.42–3.91) in the Turkish population aged 18 years and older [7]. The rhythmic, involuntary muscle activity characteristic of essential tremor is considered a primary cause of functional impairment, contributing to reduced upper-extremity control, diminished manual dexterity, and limitations in activities of daily living [8,9,10,11].

Hand tremor, one of the most common tremor types across the lifespan, is broadly categorized as resting or postural tremor, the latter emerging when a static limb position is maintained [12]. Both forms can disrupt activities of daily living and impose psychological burden, particularly when symptoms occur in social contexts. Tasks requiring fine motor control—such as handwriting, eating, dressing, and self-care—are especially affected [10,11]. Resistance training is frequently incorporated into hand tremor management programs [13], with evidence suggesting benefits for individuals exhibiting asymmetric tremor intensity [14]. Brunner et al. [15] noted that improving upper-extremity neuromuscular function in older adults may mitigate age-related decline and reduce tremor amplitude. Mechanistically, resistance exercise enhances muscle strength [16], promotes hypertrophy [17], and reduces motor unit firing variability [18] and excessive coactivation [16,19], all of which may contribute to decreased tremor amplitude. However, findings remain mixed. Budini et al. [20] reported that four weeks of manual dexterity training improved fine motor control but not tremor severity, indicating that longer or more comprehensive protocols may be necessary. In contrast, Kavanagh et al. [21] demonstrated that six weeks of high-intensity upper-extremity resistance training increased strength and improved manual dexterity in older adults with essential tremor, particularly in the more affected limb. Similar six-week resistance programs have also been shown to reduce handwriting impairments associated with essential tremor [8,22,23].

Keogh et al. [24] demonstrated that nonspecific upper-extremity strength training can enhance handgrip strength in older men, indicating that the ageing neuromuscular system remains responsive to resistance exercise. However, findings in essential tremor populations are more mixed. Bryant et al. [25] reported that a six-week home-based resistance program yielded only modest gains in handgrip strength and did not change handwriting size, whereas Park et al. [26] observed that physically active older adults exhibited significantly lower resting tremor amplitudes than sedentary individuals, suggesting a protective effect of habitual physical activity. Task-specific approaches may also be beneficial; Papale et al. [14] showed that swivel-platform hand exercises reduced tremor asymmetry, likely through enhanced neuromuscular coordination. Supporting this, Delier et al. [27] found that individuals with essential tremor had markedly lower proximal and distal muscle strength than controls, and that tremor severity was negatively correlated with strength across upper-extremity regions.

Aquatic environments offer an additional therapeutic medium due to their unique physical properties. Relative density, buoyancy, hydrostatic pressure, and thermal conductivity form the basis of aquatic therapy [28]. Buoyancy reduces mechanical loading and facilitates movement [29], hydrostatic pressure helps decrease edema, and thermal effects promote tissue relaxation and allow longer exercise tolerance in less active individuals [28,29]. These mechanisms contribute to improvements in functional capacity, joint mobility, muscle strength, and balance in older adults participating in aquatic programs [30]. Aquatic therapy has also been applied successfully in neurological conditions, including Parkinson’s disease, where it supports gait, balance, and motor control [31].

Aquatic exercise has demonstrated meaningful therapeutic benefits across various clinical populations. For instance, it has been shown to significantly improve both static and dynamic balance in individuals with multiple sclerosis and hemiplegia [32]. Polat [33] reported that recreational swimming in a natural aquatic environment (the sea) led to favorable reductions in blood pressure among older adults with stage-1 hypertension and contributed to improved metabolic safety and healthier aging. Similarly, Silva and Israel [34] found that a dual-task aquatic exercise program effectively enhanced postural control. Collectively, these findings support the growing evidence that aquatic exercise can be at least as effective as land-based training—and in some cases superior—across multiple functional domains [35].

Given that hand tremor occurs in up to 94% of individuals with essential tremor and markedly interferes with daily activities that rely on fine motor skills, the development of targeted physical activity and exercise protocols for this population remains both limited and insufficiently understood. Given the clinical heterogeneity of essential tremor, the present study specifically focused on older adults with a predominantly upper-limb action tremor phenotype. Participants were characterized by functionally relevant postural and kinetic tremor affecting daily manual activities, without additional neurological signs suggestive of ET-plus. This targeted approach was adopted to ensure a clinically homogeneous cohort and to facilitate interpretation of exercise-related effects on tremor modulation. Therefore, the present study aimed to compare the effects of (i) home-based resistance exercise and (ii) aquatic resistance exercise on upper-limb muscle strength, hand dexterity, and upper-extremity postural tremor severity in older adults with essential tremor and to determine whether the two exercise modalities differ in their impact on these outcomes.

## 2. Materials and Methods

### 2.1. Study Design

This study employed a three-arm, pretest–posttest randomized controlled trial (RCT) with participants allocated to an aquatic resistance exercise group, a home-based resistance exercise group, or a control group (3 × 2 factorial structure). The design was selected to allow direct comparison of modality-specific effects of resistance training on tremor severity and functional outcomes in older adults with essential tremor. Sample size considerations were informed by prior studies examining the effects of resistance training on tremor amplitude and upper-limb function in older adults, which generally report that 7–14 participants per group provide sufficient statistical power to detect meaningful changes [20,21,25,36]. Accordingly, the group sizes in the present trial were determined based on effect sizes reported in the literature and practical feasibility for this target population. Randomized allocation was implemented using block randomization to ensure balanced group distribution and minimize selection bias.

### 2.2. Participants

**Figure 1:**
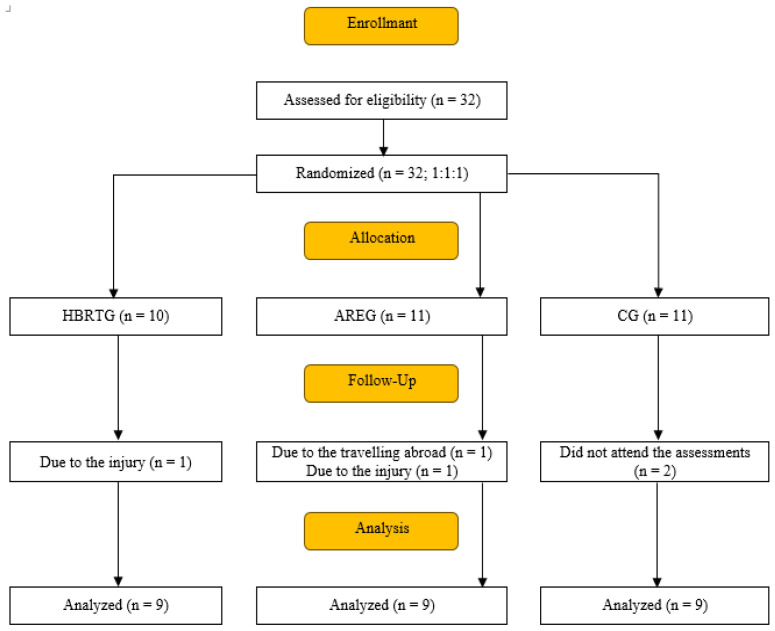
Flow diagram of participant recruitment, group allocation, follow-up, and analysis.

Older adults with essential tremor (ET) were recruited through family medicine clinics, neurology outpatient units, and physiotherapy departments in Eskişehir. Inclusion criteria were: (1) age 65–79 years, (2) neurologist-confirmed ET diagnosis based on the Movement Disorder Society consensus statement [37] and the International Tremor Foundation classification [38], and (3) no participation in regular resistance exercise within the past year. Exclusion criteria included: comorbid neurological disorders; musculoskeletal, cardiovascular, or respiratory conditions limiting safe participation; cognitive impairment (score ≤ 24) [36]; visual impairment; and use of tremor-altering medications. All participants underwent a detailed neurological examination prior to enrollment. Individuals presenting additional neurological signs suggestive of ET-plus (e.g., dystonia, parkinsonism, cerebellar signs, or cognitive impairment) were excluded. Baseline clinical presentation was characterized by predominantly upper-limb postural and kinetic tremor, with functional involvement mainly affecting daily manual activities of the dominant hand. All volunteers received written and verbal information about the study, provided informed consent, and were informed of their right to withdraw at any time. Testing procedures were conducted at the Eskişehir Technical University Biomechanics Research Laboratory. Participants were instructed to refrain from alcohol, nicotine, and caffeine on testing days. Tremor assessments included spiral drawing (Archimedes spiral), arm extension, and water pouring tasks. Two neurologists (ÖT and ŞT), blinded to group allocation, independently evaluated these tasks at pretest (week 1) and posttest (week 6) using a 0–4 scale. Ethical approval was obtained from the Scientific Research and Publication Ethics Committee of Eskişehir Technical University, Faculty of Science and Engineering (15.01.2025/E-87914408-050-62641). The study was conducted in accordance with the Declaration of Helsinki and CONSORT 2010 guidelines. A total of 32 volunteers with at least one year of ET history were initially screened; 27 participants (15 males, 12 females; mean age = 66.9 years; range = 64–76) completed the study. Participants were randomly assigned to the home-based resistance exercise group (HBREG; n = 9), the aquatic resistance exercise group (AREG; n = 9), or the control group (CG; n = 9) through block randomization performed by an independent researcher who was not involved in data collection. Figure 1 illustrates the participant flow throughout the study, including recruitment, block randomization into the home-based resistance exercise group (HBREG), aquatic resistance exercise group (AREG), and control group (CG), as well as exclusions and final numbers included in the statistical analyses.

### 2.3. Exercise Protocol

The first three exercise sessions were directly supervised by a physiotherapist to ensure correct technique and protocol familiarization. Subsequent sessions were performed independently. Adherence and exercise fidelity were monitored through weekly check-ins, during which participants were asked to report session completion, perceived exertion, and any difficulties encountered. Both intervention groups completed 18 resistance-training sessions over six weeks (three sessions per week), targeting upper-extremity muscle strength. Each session consisted of a 5 min warm-up, a 20–25 min main phase (five resistance exercises performed for three sets of 8–10 repetitions with one minute of active rest between sets), and a 5 min cool-down. Exercise intensity was standardized across groups and adjusted according to the mean RPE from the first three sessions, with a target training intensity of 4–5 au. The control group continued their usual daily routines without structured exercise. The home-based resistance exercise program consisted of five patterns: static handstand, wall angel, lateral static hold, push-ups, and biceps curls. All exercises were individually adapted to participants’ functional capacity. Exercises such as static hand stand, plank variations, and push-ups were modified as needed (e.g., wall-supported hand stands, knee-supported planks, and inclined push-ups) to ensure safety and feasibility for older adults. Progression or regression was determined based on participants’ tolerance and ability to maintain correct form. Resistance loads for upper-limb exercises (e.g., curls) were standardized using light-to-moderate resistance (elastic bands or dumbbells ranging from approximately 1–3 kg), and loads were adjusted when participants were able to complete the prescribed repetitions with proper technique. Exercise form and range of motion were standardized through initial supervised sessions, during which correct technique was demonstrated and practiced. The aquatic resistance exercise program included side-wall plank, side plank with circular arm movements plus knee curl, and the Floating Diamond Plie series (squat + forward push, torso rotation + knee lift, and reverse plank). Exercise selection for the aquatic group emphasized buoyancy-assisted stability challenges and water-based resistance. Aquatic exercise sessions were performed in a pool with water depth adjusted to the xiphoid process level and maintained at a temperature of approximately 28–30 °C. Resistance was primarily provided by water viscosity and controlled movement speed. No additional resistance equipment was used. Participants were instructed to perform movements through a comfortable and standardized range of motion, with emphasis on controlled execution to ensure consistency across sessions. The first three sessions were conducted in supervised small-group settings (HBREG in the gym; AREG in the pool). All remaining sessions were completed independently at home or in the pool, depending on group assignment. To support adherence and monitor safety, participants were contacted weekly via phone or text.

### 2.4. Data Tools

Fahn–Tolosa–Marin Tremor Rating Scale (FTMTRS): Tremor severity was assessed using the Fahn–Tolosa–Marin Tremor Rating Scale, a widely used clinical instrument consisting of three sections (A, B, and C) [10]. The assessment requires approximately 10 min and is easily comprehensible for older adults. Each item is scored on a 5-point ordinal scale ranging from 0 (no tremor) to 4 (severe tremor). The FTMTRS demonstrates strong psychometric properties, with reported inter-rater reliability of ICC ≥ 0.893 and excellent reliability for postural tremor subtests (ICC ≥ 0.953; 95% CI: 0.918–0.975; *p* < 0.001) [39]. Previous international studies have also reported excellent inter-rater internal consistency (ICC = 0.97) [11]. While inter-rater agreement ranges from fair for Part A to poor for Part B, intra-rater reliability has consistently been reported as very good across repeated assessments [19,20]. Given the aims of the present study, only Part A of the FTMTRS, which evaluates upper-extremity tremor severity, was utilized. Only Part A (tremor severity) of the FTMTRS was used in the present study to quantify tremor amplitude during specific motor tasks. Handwriting and drawing outcomes were assessed using standardized task-based spiral and line drawing tests performed independently of the FTMTRS disability subscales. These task-based measures were selected to capture fine motor performance and tremor control during functionally relevant activities. Functional performance within this section was assessed using spiral drawing and water pouring tasks, performed separately with the dominant and non-dominant hands.

Postural Hand Tremor Assessment: Postural tremor severity was evaluated using a standardized videotaped protocol. Participants were seated with hips and knees flexed at 90°, arms fully extended forward, wrists slightly extended, and fingers comfortably abducted. Tremor amplitude and severity were independently scored by a trained researcher using a 5-point scale: 0 = normal; 1 = mild tremor (<0.5 cm amplitude); 2 = moderate tremor (0.5–1 cm); 3 = marked tremor (1–2 cm); and 4 = severe tremor (>2 cm) [40].

Archimedes Spiral Test (Drawing A, B, and C): The Archimedes spiral test was used to assess tremor severity and fine motor control during a drawing task. Participants were instructed to trace spiral figures by connecting marked points without crossing boundary lines. Each hand was evaluated separately, beginning with the less affected side, and participants were instructed not to rest their hand or arm on the table to prevent external stabilization. Drawings were evaluated using a standardized 5-point scoring system: 0 = normal tracing; 1 = slight tremor with occasional boundary crossings; 2 = frequent boundary crossings; 3 = marked difficulty with multiple errors but task completion; and 4 = inability to complete the drawing due to severe tremor [40].

Pouring Task: The pouring task was used to assess functional tremor severity during a goal-directed upper-limb activity. Two hard plastic cups (8 cm in height) were used, with one cup filled to 1 cm below the rim. Participants were instructed to transfer the water from one cup to the other using each hand separately while being videotaped. Performance was scored using a standardized 5-point scale: 0 = normal performance without spillage; 1 = cautious performance without spillage; 2 = minor spillage (≤10% of total volume); 3 = moderate spillage (>10–50%); and 4 = severe impairment, characterized by inability to pour most of the water without spilling [40].

Handgrip Strength: Handgrip strength was assessed using a digital hand dynamometer (Jamar, JA Preston Co., Jackson, MI, USA). Measurements were performed in a standardized seated position with the shoulder adducted and neutral, the elbow flexed at 90°, and the forearm in a neutral position. Participants were instructed to squeeze the dynamometer maximally following a verbal cue. Each hand was tested three times with a 1 min rest interval between trials, and the mean of the three measurements was recorded as the handgrip strength value [41].

Nine-Hole Peg Test (NHPT): Manual dexterity was evaluated using the Rolyan Nine-Hole Peg Test, consisting of a molded board with nine holes and nine cylindrical pegs (0.6 cm diameter) [42]. The NHPT is a widely used, rapid, and sensitive assessment of fine motor performance, with high reported inter-rater reliability (right hand r = 0.97, left hand r = 0.99) and acceptable test–retest reliability [43]. Participants were seated with the pegboard positioned at the midline and the peg container placed on the same side as the tested hand. The dominant hand was assessed first. Following a brief demonstration, participants were instructed to insert and remove the pegs as quickly as possible using the designated hand. Timing began when the first peg was touched and ended when the final peg was returned to the container. Total completion time (seconds) was recorded for each hand [13,43].

Rating of Perceived Exertion (RPE) Scale: Perceived exercise intensity during the intervention was monitored using the session Rating of Perceived Exertion (sRPE) scale, originally developed by Borg (1988) and later adapted for session-based monitoring by Foster et al. (2001) [44,45]. This scale quantifies an individual’s subjective perception of physical exertion in response to exercise load and is widely used to regulate and standardize training intensity in clinical and exercise research.

### 2.5. Statistical Analysis

Data normality was assessed using the Kolmogorov–Smirnov test, while skewness–kurtosis values and Levene’s test were examined to evaluate variance homogeneity. Descriptive statistics were reported as median, mean rank, and 95% bias-corrected and accelerated confidence intervals (BCa CI) for the median. Within-group differences between pre- and post-intervention values were analyzed using the Wilcoxon signed-rank test. Between-group differences were examined using the Kruskal–Wallis H test based on pre–post change scores. When a significant omnibus effect was detected, pairwise comparisons were conducted using the Mann–Whitney U test with Bonferroni-adjusted significance thresholds (*p* = 0.05 ÷ 3 = 0.0167). Effect sizes for nonparametric analyses were calculated using Cohen’s r (1988) [46] criteria, interpreted as small (r = 0.10), moderate (r = 0.30), and large (r = 0.50). All statistical analyses were performed using SPSS version 25.0 (SPSS Inc., Chicago, IL, USA) [46], with significance set at *p* < 0.05. Given the modest sample size and the number of outcome measures, one primary outcome was prespecified to guide interpretation of the results. Changes in [PRIMARY OUTCOME, e.g., NHPT time or RHD-C score] were considered the primary endpoint, while all remaining outcomes were treated as secondary or exploratory. Within-group analyses were interpreted cautiously, with emphasis placed on effect sizes rather than isolated *p*-values.

## 3. Results

This section presents the statistical findings of the study. Results for the prespecified primary outcome are presented first, followed by secondary and exploratory analyses. Baseline demographic and anthropometric characteristics of the participants are summarized first (Table 1). Subsequently, within-group pre- and post-test comparisons and between-group differences in tremor severity, manual dexterity, and handgrip strength variables are reported using non-parametric statistical analyses.

**Table 1:** Baseline demographic and anthropometric characteristics of participants.

Variable	HBREG (n = 9)	AREG (n = 9)	CG (n = 9)
	M ± SD	M ± SD	M ± SD
Age (years)	70.00 ± 5.32	66.00 ± 4.87	66.00 ± 6.46
Height (cm)	171 ± 10.96	170 ± 7.66	170 ± 8.82
BW (kg)	80 ± 10.81	90 ± 12.12	76 ± 14.60
BMI (kg/m^2^)	27.4 ± 2.96	29.2 ± 4.37	27.6 ± 4.23

Values are presented as mean ± SD. Baseline comparisons between groups were performed using independent samples *t*-tests to demonstrate group comparability after randomization. No statistically significant differences were observed between any groups for age, height, body weight, or BMI (all *p* > 0.05).

Within-group Wilcoxon signed-rank analyses revealed significant pre–post improvements in several tremor-related, manual dexterity, and strength variables (Table 2). Specifically, significant differences were found for right-hand drawing-A and left-hand drawing-A in the AREG, as well as for right-hand drawing-C and left-hand drawing-C in the HBREG (Z = −2.236, *p* = 0.025, r = 0.53; Z = −2.000, *p* = 0.046, r = 0.47; Z = −2.762, *p* = 0.006, r = 0.65; Z = −2.000, *p* = 0.046, r = 0.47, respectively). These findings indicate moderate to large effect sizes for both interventions. No significant changes were observed in the drawing-B subscale for either hand. Significant within-group improvements were also observed for functional pouring tasks. Both HBREG and AREG demonstrated significant reductions in tremor severity during right- and left-hand pouring tasks (Z = −2.449, *p* = 0.014, r = 0.57; Z = −2.236, *p* = 0.025, r = 0.53; Z = −2.271, *p* = 0.023, r = 0.54; Z = −2.714, *p* = 0.007, r = 0.64), reflecting moderate to large intervention effects. With respect to manual dexterity, significant improvements were detected in the Nine-Hole Peg Test for the right hand in both intervention groups and for the left hand in the HBREG (Z = −2.668, *p* = 0.008, r = 0.63; Z = −2.670, *p* = 0.008, r = 0.63; Z = −2.668, *p* = 0.008, r = 0.63), indicating large effect sizes. Finally, dominant and non-dominant handgrip strength significantly increased in both HBREG and AREG (Z = −2.66, *p* < 0.008, r = 0.63 for all comparisons), demonstrating large intervention effects. Collectively, these results suggest that both home-based and aquatic resistance exercise programs elicited moderate to large improvements in tremor severity, manual dexterity, and handgrip strength.

According to the Kruskal–Wallis test results, significant between-group differences were observed in the pre–post change scores for RHD-A, RHD-C, LHD-A, RHP, LHP, RHNHPT, LHNHPT, DHGST, and NDHGST variables (*p* < 0.05). In contrast, no significant between-group differences were found for PHT, DHR, RHD-B, LHD-B, and LHD-C variables (*p* > 0.05). These findings indicate that the intervention type had a differential effect on manual dexterity and handgrip strength outcomes (Table 3).

According to the Bonferroni-adjusted post hoc Mann–Whitney U test results, significant between-group differences were observed for several variables following the Kruskal–Wallis analysis (Table 4). Specifically, for the RHD-A variable, a significant difference was found between the AREG and CG (*p* = 0.011, r = 0.49). For the RHD-C variable, significant differences were observed between the HBREG and CG (*p* = 0.001, r = 0.92) as well as between the HBREG and AREG (*p* = 0.001, r = 0.90), indicating large effect sizes. In addition, for the LHD-A variable, a significant difference was detected between the HBREG and CG (*p* = 0.029, r = 0.51), while no significant differences were found for the remaining pairwise comparisons after Bonferroni correction (*p* > 0.0167). Overall, these findings suggest that both intervention types resulted in meaningful improvements compared to the control group for selected tremor-related and manual dexterity outcomes, with home-based resistance exercise demonstrating relatively stronger effects in selected variables. Accordingly, only pairwise comparisons meeting the Bonferroni-adjusted significance threshold (*p* < 0.0167) were considered statistically significant. Dropouts in both intervention groups were related to minor musculoskeletal complaints. These events were mild in severity, did not require medical intervention, and were not clearly attributable to the exercise protocols. No serious adverse events were reported.

## 4. Discussion

Significant differences observed between the home-based resistance exercise group and both the control and aquatic exercise groups on the RHD-C subscale indicate that home-based resistance training may produce a stronger effect on the directional stability of tremor. This finding suggests that land-based resistance exercise provides more task-specific neuromuscular loading, facilitating improvements in directional motor control during fine motor tasks. These findings are consistent with previous studies reporting that resistance-based interventions effectively reduce tremor amplitude and severity, particularly in tasks requiring continuous proprioceptive feedback and motor adaptation, such as manual dexterity tasks [2,8,10,11,14,15,16,17,18,19,20,21,22,23]. The repetitive and controlled nature of resistance exercise may enhance sensorimotor integration and reinforce central motor planning processes, thereby strengthening the directional components of motor control. Although aquatic resistance exercise also demonstrated a regulatory effect on tremor severity, its impact on directional stability appeared comparatively less pronounced. This difference may be explained by water-induced damping and reduced gravitational loading, which may attenuate tremor amplitude while providing less directional challenge during task execution. Furthermore, the absence of significant improvements in the RHD-B and LHD-B drawing tasks suggests task-specific motor demands. These tasks may impose higher visuomotor integration and hand–eye coordination requirements, which are more sensitive to exercise intensity, volume, or intervention duration. This observation indicates that more complex drawing tasks may require longer training periods or more targeted coordination-focused interventions to elicit measurable improvements. Collectively, these findings contribute to the literature by demonstrating that while both resistance exercise modalities are effective for tremor regulation, home-based resistance exercise may offer superior benefits for directional tremor control during fine motor tasks in older adults with essential tremor.

According to the pretest–posttest difference analysis, significant between-group differences were observed in selected right- and left-hand drawing and pouring outcomes, indicating that resistance-based exercise interventions produced modality-specific effects on tremor regulation. Post hoc analyses demonstrated that home-based resistance exercise elicited moderate improvements compared with the control condition across both right- and left-hand outcomes, reflecting a consistent effect on directional motor control. These findings are in line with previous studies reporting that land-based resistance training can reduce tremor amplitude and improve fine motor control through enhanced neuromuscular coordination and sensorimotor integration [8,14,20,21]. In contrast, aquatic resistance exercise showed a more pronounced effect on left-hand performance, with larger effect sizes observed in non-dominant hand tasks. Similar observations have been reported in neurological and aging populations, where aquatic exercise preferentially improved movement regulation and coordination in less-dominant limbs due to water-induced damping and increased proprioceptive input [31,32,33,34,35]. The marked improvement in left-hand pouring performance is particularly noteworthy, as it suggests that tremor regulation may be more responsive to intervention in the non-dominant limb. This phenomenon may be explained by greater neural plasticity or a higher potential for neuromuscular adaptation in less frequently utilized motor patterns, as previously suggested in motor learning and aging research [18,22,27]. Collectively, these findings indicate that both resistance-based exercise modalities positively influence hand–eye coordination and fine motor control, while differing in their specific motor adaptations. Home-based resistance exercise appears to provide more consistent improvements in directional tremor stability, whereas aquatic resistance exercise may confer additional benefits for non-dominant hand tremor regulation. These modality-specific adaptations extend previous work by demonstrating that exercise environment plays a role in shaping tremor-related motor outcomes in older adults with essential tremor.

In contrast, the absence of significant improvements in the control group underscores the specificity of the observed adaptations to the applied exercise interventions rather than to spontaneous recovery or test–retest effects. Consistent with these findings, our previous studies have demonstrated that structured and recreational exercise interventions can induce significant improvements in musculoskeletal strength and functional performance in older adults, supporting the notion that appropriately designed exercise modalities contribute to neuromuscular adaptations beyond spontaneous recovery effects. Moreover, the positive outcomes detected in both exercise modalities appear to be closely related to the significant improvements observed in the Nine-Hole Peg Test, a task that integrates handgrip strength, fine motor precision, and sensorimotor coordination. From a clinical perspective, even modest improvements in manual dexterity and functional task performance may translate into greater ease during everyday activities such as eating, writing, and personal grooming. Such functional gains can reduce activity-related frustration and enhance confidence in social situations, which are highly relevant outcomes for individuals living with essential tremor. These observations are supported by previous studies examining the effects of resistance-based exercise interventions in individuals with essential tremor and Parkinson’s disease [29,47,48,49,50,51,52,53,54,55,56], which have consistently reported enhancements in manual dexterity, grip strength, and tremor regulation following structured resistance training programs.

Improvements observed in manual dexterity tasks indicate that resistance-based exercise interventions positively influence fine motor performance in older adults with essential tremor. Home-based resistance exercise was associated with more pronounced improvements in manual dexterity, whereas aquatic resistance exercise also elicited meaningful, albeit more moderate, gains. These findings suggest that resistance training performed in different environments may differentially affect fine motor control. Previous studies have demonstrated that resistance and coordination-focused training can enhance manual dexterity by improving neuromuscular coordination, motor unit control, and sensorimotor integration in both aging and neurological populations [8,13,20,21,43]. In particular, land-based resistance exercise has been shown to support improvements in task precision and execution speed, which are critical components of Nine-Hole Peg Test performance. The beneficial effects observed following aquatic resistance exercise may be attributed to the unique properties of the water environment, including increased viscosity and buoyancy, which can facilitate smoother movement execution and enhance proprioceptive feedback. Such mechanisms have been previously reported to support improvements in upper-limb coordination and fine motor control during aquatic interventions [31,32,33,34,35]. Collectively, these results extend existing evidence by demonstrating that both home-based and aquatic resistance exercise modalities can effectively improve manual dexterity in older adults with essential tremor, while highlighting potential modality-specific adaptations related to exercise environment.

Hand tremor represents the most prevalent manifestation of essential tremor in older adults and, when combined with age-related morphological and physiological changes, imposes considerable limitations on daily functional performance. Among the key determinants of functional independence and quality of life in this population are fine manipulative hand skills. The results of the present study demonstrate that both exercise modalities positively influenced fine motor dexterity and contributed to a reduction in tremor severity, despite the relatively short intervention duration. These findings are consistent with previous research reporting beneficial effects of resistance-based exercise interventions on manual dexterity, grip strength, and tremor control in individuals with essential tremor and Parkinson’s disease [4,8,13,20,21,57]. The findings of the present study demonstrate that both home-based and aquatic resistance exercise interventions significantly improved dominant and non-dominant handgrip strength in older adults with essential tremor, with comparable effect sizes observed for the two exercise modalities. These strength gains are likely attributable primarily to neural and functional adaptations rather than morphological changes, particularly given the relatively short intervention duration. Such adaptations may include increased motor unit recruitment and firing rates, enhanced motor unit synchronization, and improved coordination between agonist and antagonist muscle groups in the upper extremities of individuals with essential tremor [4,8,14,16,17,18,19,21,22,23,27,28,29].

Improvements observed in functional tasks, such as pouring performance and Nine-Hole Peg Test outcomes, may be explained by concurrent enhancements in intramuscular and intramuscular coordination. Resistance-based exercises that incorporate fine motor components can facilitate improvements in manual dexterity and finger range of motion; however, the present findings also suggest that more targeted, task-specific interventions focusing explicitly on manual dexterity may be required to optimize functional outcomes. In this regard, systematic load progression in accordance with the principle of progressive overload may further enhance neuromuscular adaptations and yield more comprehensive improvements in functional performance. Overall, these results are consistent with previous literature examining the effects of resistance exercise performed in different environments on muscle strength, manual dexterity, and functional capacity in older adults with essential tremor and related neurological conditions [31,35,41,48,49,51,55,57]. Collectively, the evidence supports the use of resistance-based exercise interventions as an effective strategy for improving upper-extremity strength and functional hand performance in this population. The relatively small sample size and short intervention duration may limit the generalizability of the findings and the interpretation of complex visuomotor outcomes. Additionally, the lack of long-term follow-up prevents conclusions regarding the sustainability of the observed improvements. A further limitation of this study is that formal inter-rater reliability statistics were not calculated for video-based assessments, although consensus ratings were used to minimize rater-related variability. Secondary and exploratory findings should be interpreted with caution given the number of outcomes assessed and the limited sample size. In addition to the small sample size and short intervention duration, the absence of an active control condition should be considered when interpreting the findings. Participants were aware of their group allocation, which may have introduced expectancy- or attention-related effects. Although outcome assessments were performed by blinded raters, future studies should incorporate an active comparator (e.g., stretching or education-based programs), longer follow-up periods, and objective tremor quantification methods such as wearable sensors or accelerometry to better distinguish true physiological changes from contextual effects.

## 5. Conclusions

The findings of this randomized controlled trial demonstrate that both home-based and aquatic resistance exercise interventions effectively reduce tremor severity and improve manual dexterity and handgrip strength in older adults with essential tremor. Significant improvements were observed in tremor-related drawing and pouring tasks, Nine-Hole Peg Test performance, and dominant as well as non-dominant handgrip strength, whereas no meaningful changes were detected in the control group. These results highlight the specific and beneficial effects of structured resistance-based exercise programs on upper-extremity motor function in this population. Importantly, no significant differences were identified between the two exercise modalities across most outcome measures, suggesting that both home-based and aquatic resistance exercises represent viable and effective intervention strategies. However, home-based resistance exercise demonstrated particularly strong effects on directional tremor stability in selected tasks, while aquatic resistance exercise appeared to confer additional benefits for non-dominant hand performance. These modality-specific tendencies may reflect differences in neuromuscular loading, proprioceptive input, and movement regulation associated with land-based versus aquatic environments. From a clinical perspective, the present findings support the integration of resistance-based exercise programs into the management of essential tremor in older adults. Home-based interventions offer a cost-effective and accessible option, particularly for individuals with limited access to specialized facilities, whereas aquatic resistance exercise may provide an alternative for those requiring reduced joint loading or enhanced movement support. Together, these approaches have the potential to improve functional independence, self-care ability, and quality of life in older adults affected by essential tremor. Future studies should investigate the long-term effects of resistance-based exercise, explore dose–response relationships related to exercise intensity and progression, and examine the impact of more task-specific or coordination-focused training protocols on complex visual motor tasks. Such research will further refine exercise prescription strategies and optimize functional outcomes for individuals with essential tremor. Nevertheless, the present findings should be interpreted in light of certain limitations, including the relatively small sample size, short intervention duration, and the absence of long-term follow-up.

## Figures and Tables

**Table 2:** Within-group pre- and post-test comparisons of tremor severity, manual dexterity, and strength variables (Wilcoxon signed-rank test).

Variables	Groups	Z	*p*	r
PHT	HBREG (n = 9)	−1.000	0.317	0.24
	AREG (n = 9)	−1.414	0.157	0.33
	CG (n = 9)	0.000	1.000	0.00
Handwriting	HBREG (n = 9)	−1.000	0.317	0.24
	AREG (n = 9)	−1.000	0.317	0.24
	CG (n = 9)	0.000	1.000	0.00
RHD-A	HBREG (n = 9)	−1.890	0.059	0.45
	AREG (n = 9)	−2.236	0.025	0.53
	CG (n = 9)	0.000	1.000	0.00
RHD-B	HBREG (n = 9)	−1.732	0.083	0.41
	AREG (n = 9)	−1.633	0.102	0.38
	CG (n = 9)	−1.414	0.157	0.33
RHD-C	HBREG (n = 9)	−2.762	0.006	0.65
	AREG (n = 9)	−1.000	0.137	0.24
	CG (n = 9)	0.000	1.000	0.00
LHD-A	HBREG (n = 9)	−1.633	0.102	0.38
	AREG (n = 9)	−2.00	0.046	0.47
	CG (n = 9)	−1.414	0.157	0.33
LHD-B	HBREG (n = 9)	−1.732	0.083	0.41
	AREG (n = 9)	−1.732	0.083	0.41
	CG (n = 9)	−1.000	0.317	0.24
LHD-C	HBREG (n = 9)	−2.000	0.046	0.47
	AREG (n = 9)	−1.732	0.083	0.41
	CG (n = 9)	−1000	0.317	0.24
RHP	HBREG (n = 9)	−2.449	0.014	0.57
	AREG (n = 9)	−2.236	0.025	0.53
	CG (n = 9)	−0.577	0.564	0.14
LHP	HBREG (n = 9)	−2.271	0.023	0.54
	AREG (n = 9)	−2.714	0.007	0.64
	CG (n = 9)	0.000	1.000	0.00
RHNHPT	HBREG (n = 9)	−2.668	0.008	0.63
	AREG (n = 9)	−2.670	0.008	0.63
	CG (n = 9)	−0.416	0.678	0.10
LHNHPT	HBREG (n = 9)	−2.668	0.008	0.63
	AREG (n = 9)	−1.601	0.109	0.38
	CG (n = 9)	−1.483	0.138	0.35
DHGS	HBREG (n = 9)	−2.666	0.008	0.63
	AREG (n = 9)	−2.666	0.008	0.63
	CG (n = 9)	−1.836	0.066	0.43
NDHGS	HBREG (n = 9)	−2.666	0.008	0.63
	AREG (n = 9)	−2.666	0.008	0.63
	CG (n = 9)	−1.481	0.139	0.35

Note: Z represents the Wilcoxon signed-rank test statistic, *p* indicates the level of statistical significance, and r denotes the effect size. Effect size interpretation according to Cohen (1988) [46]: small effect (r < 0.30), moderate effect (0.30 ≤ r < 0.50), and large effect (r ≥ 0.50). Abbreviations: HBREG, Home-Based Resistance Exercise Group; AREG, Aqua Resistance Exercise Group; CG, Control Group; PHT, Postural hand tremor; RHD-A, Right hand drawing–A; RHD-B, Right hand drawing–B; RHD-C, Right hand drawing–C; LHD-A, Left hand drawing–A; LHD-B, Left hand drawing–B; LHD-C, Left hand drawing–C; RHP, Right hand pouring; LHP, Left hand pouring; RHNHPT, Right-hand Nine-Hole Peg Test; LHNHPT, Left-hand Nine-Hole Peg Test; DHGS, Dominant hand grip strength; NDHGS, Non-dominant hand grip strength. RHD, right-hand drawing; LHD, left-hand drawing.

**Table 3:** Between-group comparisons of pre–post change scores (Δ) for tremor severity, manual dexterity, and strength variables (Kruskal–Wallis test).

Variables	H	*p*
PHT	2.00	0.36
DHR	2.89	0.23
RHD-A	7.387	0.039 *
RHD-B	0.439	0.80
RHD-C	23.58	0.001 *
LHD-A	7.387	0.025 *
LHD-B	3.957	0.138
LHD-C	3.957	0.138
RHP	7.62	0.022 *
LHP	13.107	0.001 *
RHNHPT	12.158	0.002 *
LHNHPT	12.47	0.002 *
DHGST	18.137	0.001 *
NDHGST	17.365	0.001 *

Note: Δ indicates pre–post change scores. H represents the Kruskal–Wallis test statistic. Between-group differences were analyzed using the Kruskal–Wallis H test. * Statistically significant difference between groups (*p* < 0.05). DHR, dominant hand regulation; RHDNDHR, right-hand drawing; LHD, left-hand drawing.

**Table 4:** Bonferroni-adjusted post hoc pairwise comparisons (Mann–Whitney U test) for variables showing significant Kruskal–Wallis results.

Değişkenler	Gruplar	U	Z	*p*	r
RHD-A	HBREG-CG	22.00	−2.191	0.042	0.52
	HBREG-AREG	38.50	−0.199	0.084	0.04
	AREG-CG	18.00	−2.557	0.011	0.49
RHD-C	HBREG-CG	0.000	−3.912	0.001	0.92
	HBREG-AREG	0.000	−3.827	0.001	0.90
	AREG-CG	36.00	−1.000	0.317	0.23
LHD-A	HBREG-CG	21.00	−2.183	0.029	0.51
	HBREG-AREG	27.00	−0.199	0.084	0.04
	AREG-CG	18.00	−1.837	0.066	0.43
RHP	HBREG-CG	15.00	−2.491	0.013	0.58
	HBREG-AREG	36.00	−0.470	0.063	0.09
	AREG-CG	18.50	−2.182	0.029	0.51
LHP	HBREG-CG	13.50	−2.863	0.004	0.67
	HBREG-AREG	36.50	−0.406	0.685	0.08
	AREG-CG	4.500	−3.618	0.001	0.85
RHNHPT	HBREG-CG	4.500	−3.184	0.001	0.75
	HBREG-AREG	22.00	−1.637	0.101	0.38
	AREG-CG	14.50	−2.304	0.021	0.54
LHNHPT	HBREG-CG	2.50	−3.359	0.001	0.79
	HBREG-AREG	27.00	−1.195	0.232	0.28
	AREG-CG	13.50	−2.387	0.017	0.56
DHGST	HBREG-CG	0.000	−3.576	0.001	0.84
	HBREG-AREG	24.00	−1.457	0.145	0.34
	AREG-CG	0.000	−3.576	0.001	0.84
NDHGST	HBREG-CG	0.000	−3.576	0.001	0.84
	HBREG-AREG	39.00	−0.132	0.895	0.03
	AREG-CG	0.000	−3.576	0.001	0.84

Post hoc comparisons were performed using the Mann–Whitney U test following significant Kruskal–Wallis results. Bonferroni correction was applied and the adjusted significance level was set at *p* < 0.0167.

## Data Availability

The data obtained in this study are presented in tables within the article. The raw data are available from the corresponding author upon reasonable request. The data are not publicly available due to ethical and privacy restrictions.
